# Formatively assessing prospective teachers’ skills in leading mathematics discussions

**DOI:** 10.1007/s10649-021-10070-z

**Published:** 2021-07-01

**Authors:** Meghan Shaughnessy, Nicole M. Garcia, Michaela Krug O’Neill, Sarah Kate Selling, Amber T. Willis, Charles E. Wilkes, Sabrina Bobsin Salazar, Deborah Loewenberg Ball

**Affiliations:** 1 Wheelock College of Education and Human Development, Boston University, Two Silber Way, Boston, MA 02215 USA; 2 University of Michigan, Ann Arbor, MI 48109 USA; 3 Stanford University, Stanford, CA 94305 USA; 4 Universidade Federal de Pelotas, Pelotas, RS 96010-610 Brazil

**Keywords:** Teacher education, Mathematics discussion, Assessment, Practice-based teacher education, Prospective teachers

## Abstract

Mathematics discussions are important for helping students to develop conceptual understanding and to learn disciplinary norms and practices. In recent years, there has been increased attention to teaching prospective teachers to lead discussions with students. This paper examines the possibilities of designing a formative assessment that gathers information about prospective elementary teachers’ skills with leading problem-based mathematics discussions and makes sense of such information. A decomposition of the practice of leading discussions was developed and used to design the assessment. Nine first-year teachers who graduated from a range of different teacher education programs participated in the study. The findings reveal that our formative assessment works to gather information about teachers’ capabilities with leading discussions and that the associated tools support making sense of the information gathered. This suggests that such tools could be useful to support the formative assessment of the developing capabilities of prospective teachers.

Discussions can support students in developing a conceptual understanding of mathematics (e.g., Michaels et al., 2008) and learning disciplinary norms and practices (e.g., Lampert, 2001; Yackel & Cobb, 1996). Further, discussions can broaden conceptions of what it means to know and do mathematics past the typical boundaries of being fast and correct. Broadened conceptions can include mathematical literacy skills such as speaking, listening, critiquing, and representing (Hoffer, 2016; Shanahan & Shanahan, 2008), allowing students with broad ranges of skills to see themselves as mathematically capable (Solomon, 2009). Discussions can also open up space for students to engage in participation in mathematical discourse, in mathematical practices, and opportunities to build mathematical proficiency, which together constitute academic literacy in mathematics (Moschkovich, 2015). This complex view of mathematics means that rich discourse relies on more than spoken and written language. Rich discourse also entails multiple representations and ways of communicating ideas, including social mathematical language, home language, and everyday language (Moschkovich, 2015). Through such discourse, students negotiate meanings of mathematical concepts (Smith III et al., 1994) and are positioned as capable doers of mathematics. How teachers facilitate discussions with students is consequential for students’ experiences, mathematics learning, and identity development (Aguirre & Martin, 2013; Bartell et al., 2017). Our conceptualization of discussion draws on a theory of teaching as interactive and situated in contexts (Cohen et al., 2003; Lampert, 2001) and, in particular, focused on the development of students’ ability to interact with others and with the mathematics. This study is grounded in the explicit acknowledgment of the importance of the teacher’s role in supporting students’ engagement and learning through discussion.

We believe that formative assessment could make a substantial contribution to the preparation of new teachers to lead discussions with children. By formative assessment, we mean assessments used in the flow of teacher preparation (not at its conclusion) to formulate subsequent learning opportunities (Cizek, 2010). Formative assessment enhances learning by revealing the current state of learners’ knowledge, skills, and dispositions and ensuing action that facilitates growth (Black & Wiliam, 1998; Hattie & Timperley, 2007; Shute, 2008). We use the term skills to describe prospective teachers’ (PTs) abilities to carry out specific aspects of the work of teaching at a particular moment in time, fully recognizing that teachers’ capabilities will grow and change over time. Studies of the development of expertise have found that practice opportunities alone do not sufficiently support novices to improve. Practice opportunities need to be coupled with structured directive coaching (Ericsson & Pool, 2016). Thus, formative assessment is a critical component in teacher preparation (Association of Mathematics Teacher Educators, 2017; Darling-Hammond et al., 2005; Grossman, 2009). However, many current assessments of teaching used in the USA, including observation and portfolios, measure PTs’ skills with broad domains of work such as planning, instruction, or assessment. We need additional tools to assess PTs’ skills with particular practices and components of teaching (Shaughnessy et al., 2018; Shaughnessy & Boerst, 2018). We argue that if we develop and refine formative assessments of teaching practice and tools that support teacher educators in providing timely feedback to PTs on their skills, then PTs will develop more robust skills with these teaching practices, impacting student learning of mathematics. Because our view of the teaching is that it is interactive and situated in contexts, tools that support noticing PTs’ skills with teaching practice and, in turn, providing feedback to PTs must focus on the interactive work of teaching and be responsive to the contexts in which the teaching is being enacted.

We explore a means to examine the skills PTs are developing for leading a discussion with children to focus our efforts to support PTs’ development. Leading a discussion is complex. It is interactive, and success is contingent upon skills with other teaching practices, such as implementing norms for classroom discourse. To address this need, we sought to investigate whether it would be feasible to design a formative assessment focused on leading mathematics discussions for use with prospective elementary teachers. Specifically, we investigated whether such an assessment could elicit and reveal detailed aspects of teachers’ discussion-leading skills.

## The teaching practice of leading mathematics discussions

### Mathematics discussions

The importance of mathematics discussions has been well established. It has also been established that simply having students share ideas in a “show and tell” format is not sufficient to create the type of rich collective thinking that can support student learning (Kazemi & Stipek, 2001). Teachers must encourage and value student ideas and use them to shape classroom discourse in productive ways (Sherin, 2002; White, 2003). Further, teachers must be able to notice instances of students’ mathematical thinking that have high potential to support students in understanding core mathematical ideas if discussed further (Van Zoest et al., 2017). The mathematical point of the discussion must be identified, and teachers must “steer instruction” towards this point (Sleep, 2012). Teachers must also balance different ways of participating, recognizing that there are ways to participate silently in a discussion (Moschkovich, 2015, 2018) that have been shown to support student’s learning of content (O’Connor et al., 2017) and that what it means to participate in a discussion is shaped by cultural contexts (Xu & Clarke, 2019). Importantly, the actions of teachers inside of a mathematics discussion are dependent on where the discussion is situated within a longer sequence of work (Ponte & Quaresma, 2016). This paper focuses on problem-based mathematics discussions where students engage with a single task or a set of connected tasks before participating in a discussion. The focus of the discussion itself can vary. For example, it could be focused on coming to a consensus on the answer to a mathematics problem, or it could be focused on understanding a set of different approaches that could all be used to arrive at the same answer. We focus on problem-based discussions because they are the primary sort of discussion in schools in the USA and they are frequently a focus of work in teacher education.

### Decomposing the teaching practice

From our perspective, a classroom discussion is a period of relatively sustained dialog among the teacher and multiple members of the class in which students respond to and use one another’s ideas to develop collective understanding. To assess capabilities in leading discussions, we drew upon Grossman et al.’s (2009) notion of parsing teaching practice into specific areas of work to create a “decomposition” of the practice. Because we were interested in using decomposition in teacher preparation, we attended to the importance of decomposing in ways that the practice can be taught and learned by PTs (Boerst et al., 2011). We drew upon prior research on the importance of discussion for student learning and practices for orchestrating discussion to develop an unpacking of the work of leading a discussion, a “decomposition” of the teaching practice. We identified different aspects of what teachers have to do to lead a discussion. We acknowledge that a decomposition is a living document. The decomposition can be elaborated through its use, learning about what it supports noticing and what seems salient but is not captured in the decomposition, and the decomposition can be elaborated as our field’s understanding of the teaching practice of leading a discussion grows. For example, as the field learns more about the most productive types of mathematical contributions made by teachers during discussions, detail might be added naming more specific types of contributions. One challenge of decompositions is that while they are meant to provide details about the work of carrying out the practice, they cannot name the complete set of knowledge and skills required to carry out the practice or they will be unwieldy. While we recognize that teaching practice is dependent on mathematical knowledge for teaching and teachers’ views of mathematics and children’s ideas, the decomposition is focused squarely on teachers’ enactment of the teaching practice. We return to the idea of the decomposition being a living document in the discussion.

Our decomposition deliberately foregrounds certain aspects of the work of leading discussions with students––specifically those that we consider crucial for entry-level teaching. Distinctive in our effort to articulate the work of discussion leading was that we kept in focus what is necessary for quality beginning teaching. We further considered the aspects of leading discussions that are most likely to be accessible to and learnable by novices. We began by identifying specific aspects of leading discussions, rooted in explicit orientations and purposes, and what teachers of any level of experience and expertise do to try to accomplish those. For example, in orchestrating classroom discussions, one important goal is to support students to listen to and build on one another’s ideas. This entails specific moves that can encourage students to orient their talk to one another (e.g., Chapin et al., 2013; Lampert, 2001). Another goal is to help students explain their thinking, including analyzing apparent mistakes and synthesizing and revoicing others’ thinking (Erath, 2018; Rougée, 2017). Moves that support these goals should be seen in a mathematics discussion, regardless of the level of experience of the teacher because they are crucial for student learning. These moves are also foundational for more complex work that experienced teachers do in mathematics discussions, such as supporting students to make connections across multiple contributions. These more advanced moves are not included in the decomposition because while they are important, we do not find them to be foundational to the work of leading a discussion.

The field’s understanding of classroom discussions has been enriched by a large body of research, including research focused on student–teacher relationships (e.g., Battey et al., 2016), discourse (e.g., Herbel-Eisenmann & Wagner, 2010), and participation supports and structures (e.g., Cohen & Lotan, 2014; Esmonde, 2009; Featherstone et al., 2011; Foote & Lambert, 2011; Hunter & Hunter, 2018). Our work was informed by others’ research but filtered through our particular goals of developing a decomposition of leading discussions useful for beginning teaching. For example, Reinholz and Shah (2018) carefully analyze the patterns of participation in classrooms and show the complexity of managing who gets the floor and commitments to equity. Whereas they do not identify specific things teachers can do to manage that complexity, in the area of work that we call “orchestrating,” we include deliberate attention to the moves teachers can make to distribute turns of talk in whole group discussions. Others (e.g., Chapin et al., 2013; Herbel-Eisenmann et al., 2013) have identified specific talk moves that teachers might use during a discussion (e.g., Could you add onto [student’s name] idea?). We have drawn upon this work in our decomposition and tried to explicitly connect these moves to the purposes that teachers might have for using them. These talk moves are embedded inside of our decomposition.

In our decomposition, we distinguish between discussion-enabling practices and discussion-leading practices (T. Boerst, personal communication, September 15, 2009). Discussion-enabling practices are not part of the discussion but are crucial for skillfully leading a mathematics discussion. For example, selecting a task occurs before a discussion, but selecting a discussable mathematics task is critical for having a productive discussion (Moschkovich, 2015; Smith & Stein, 1998; Ponte & Quaresma, 2016). The work that a teacher does to get students ready to engage with the task impacts what both the teacher and students can accomplish during the discussion (Jackson et al., 2012; Jackson et al., 2013). Other examples include establishing goals, anticipating student thinking about the task, and monitoring as students work on the task (Smith & Stein, 2011).

**Fig. 1:**
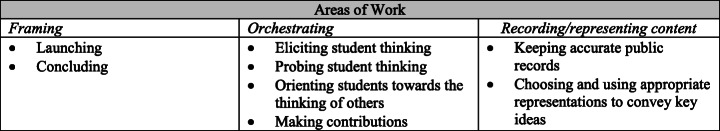
Areas of work for discussion-leading practices

Discussion-leading practices are practices used to carry out a discussion, including *eliciting student thinking*, *making contributions*, *orienting students towards the thinking of others*, *keeping accurate public records*, *using representations to convey key ideas*, and *concluding*. We organized these practices into three areas of work: framing, orchestrating, and recording/representing content (see Fig. 1). For each of the practices within an area of work, we further identify specific things that teachers can do. We refer to these as moves. There is some sequentiality implied in these areas of work because framing–launching, orchestrating, and framing–concluding occur in a specific sequence. Recording/representing content may occur throughout a discussion. Specific moves within discussion-leading practices do not need to occur in a specific sequence, although in practice, some naturally occur before others.

Framing refers to *launching* and *concluding* a discussion. In other words, the work done by a teacher to get students into a mathematics discussion and helping students make sense of what to take away from a mathematics discussion. *Launching* is important for supporting students’ productive participation in a discussion and concluding supports the transfer of learning (Engle, 2006; Engle et al., 2012). Specific moves that teachers may use to launch a discussion include “getting the attention of the class” and “directing attention towards the mathematics intended for the discussion.” *Launching* a discussion is separate from the setting up of the task (often referred to as a launch in the launch–explore–explain lesson structure). Different moves are used to *conclude* a discussion, including “making a closing statement that indicates that the discussion is ending” and “supporting students in remembering or making sense of at least one key mathematical idea or practice.”

Orchestrating, the core area of work, refers to a sustained time in which students are sharing ideas and responding to and building on one another’s ideas. Teachers orchestrate a discussion using practices such as *eliciting contributions*, *probing student thinking*, *orienting students to thinking of others*, and *making contributions*. *Eliciting contributions* includes inviting student participation with a focus on eliciting multiple solutions, methods, or strategies (Herbel-Eisenmann et al., 2013). *Probing student thinking* refers to the work that teachers do to follow on individual students’ thinking to get students to further elaborate their ideas (Herbel-Eisenmann et al., 2013), including actions that teachers can use to ask for clarification and elaboration (Erath, 2018; Prediger & Pöhler, 2015; Prediger et al., 2015), extend student thinking (Cengiz et al., 2011), and make communicative demands explicit (Erath, 2018). *Orienting students to thinking of others* refers to the work that teachers do to support students in engaging with others’ ideas, including “asking students to revoice (or restate) another’s idea” (Chapin et al., 2013; Herbel-Eisenmann et al., 2013), “posing questions to students about others’ ideas and contributions” (Herbel-Eisenmann et al., 2013), and “encouraging students to attend, listen and respond to peers’ contributions.” Such engagement supports student learning, and the teacher has a critical role in supporting this engagement (Franke et al., 2015). *Making contributions* refers to the direct contributions that teachers make to a discussion, such as “ensuring that substantive and relevant analysis is part of the discussion” or “introducing particular vocabulary” (a mathematical contribution). Each of these orchestrating practices also includes moves required for carrying out the practice. For example, moves for *orienting* include (a) “posing questions to students about others’ ideas and contributions,” (b) “supporting the listening of the class through the use of moves that require all students to respond to others’ work,” and (c) “encouraging students to attend, listen and respond to peers’ contributions in order to maintain productive and focused interaction.”

Further, in discussions, teachers *record and represent ideas* in ways that support learners to engage in the discussion. Within the discussion itself, making public records of the collective mathematical work supports collective sensemaking (Garcia et al., n.d.; Ball & Bass, 2003). Alternatively, they might support their students in doing the representing and recording work. We refer to this area of work as recording and representing content. We distinguish between two specific practices, each of which includes particular moves: (1) *keeping accurate records* and (2) *choosing and using appropriate representations to convey key mathematical ideas*.

We acknowledge that our decomposition of the teaching does not include all of the specific skills and considerations necessary for supporting productive discussions. For example, to engage students with others’ ideas, teachers must also support students in learning what it means to participate in discussions in ways that are respectful of how students interact in their communities outside of school (Hunter & Hunter, 2018). This might include, for example, explicitly teaching students what it means to challenge someone’s idea in a math class. Another example is that successful discussions are dependent upon the relationships that have been developed between the teacher and students and among students. Thus, teachers must be able to build such relationships with and between students. Yet another example not named in the decomposition is the work of ensuring equitable participation in the discussion. Teachers must be able to monitor who is participating and intentionally support the participation of a broad range of students. The bounding of the work of teaching is a major challenge in developing a decomposition of teaching practice.

We believe that the decomposition of discussion leading that we developed contributes usefully to the design of learning experiences for teachers because it highlights specific skills and considerations necessary in conducting mathematics discussions with students. It also provides a framework for formatively assessing PTs’ evolving capabilities. We now turn to the assessment of PTs’ skills.

### Assessing prospective teachers’ discussion-leading practice

The design of the formative assessment was influenced by our view of teaching as interactive and situated in contexts (Cohen et al., 2003; Lampert, 2001) as well as our goals of using information elicited in formative ways. Our view of teaching suggests that as teachers lead discussions, the students, the content, and the environment matter. For example, a teacher’s knowledge of the content, the “discussability” of the task, students’ prior experiences participating in discussions, and the teacher’s knowledge of and relationships with their students shape how discussions play out (Boerst et al., 2011). This led us to identify two considerations in the design of the assessment. First, to support student learning, a teacher must make moves in the moment that are responsive to their students and the context in which they are teaching. By this, we mean that the moves that a teacher makes during the discussion cannot be scripted. The moves made by the teacher need to be responsive to the ideas shared by students, the ways in which those ideas relate to the mathematics of the task, and the resources brought by specific individuals in the class. This led us to situate our assessment in classrooms where teachers had relationships with students and where teachers were positioned to understand and be responsive to the context in which they were teaching. Second, it was also important that the assessment acknowledges variations in classroom norms and grade levels and not prescribe teaching practice in a way that fails to account for the diversity of resources that children and teachers bring, how teachers and students interact together, and developmental differences such as differences in the ways in which five-year-old children and ten-year-old children participate in discussions.

Further, a set of three considerations arose from the focus of the assessment and how we wanted to use the knowledge gathered. First, the enactment of leading a discussion matters. Thus, we set out to design an assessment focused squarely on their ability to carry out discussions in real time. Second, teachers’ knowledge of the content, the “discussability” of the task, and their skills in getting students to work on a task before a discussion shape how discussions play out (Boerst et al., 2011). These factors can make it difficult to elicit PTs’ skills for leading a discussion. For example, when a teacher leads a discussion around a task that is not well-suited for a discussion, the enactment often does not capture the skills with leading a discussion that the teacher has developed. Thus, we chose to provide the task, supports for unpacking the mathematics of the task, and detailed instructions about how to introduce the task and have the students work on the task prior to the discussion. These supports fell within the space of discussion-enabling work. Third, we sought to have our assessment provide fine-grained detail about the demonstrated skills of individuals in order to support their development. We also sought to capture skills at a grain size that did not specify a particular language that PTs must use so that PTs could be responsive to the children.

With these goals and the decomposition of discussion in mind, we developed a formative assessment to be implemented in PTs’ classrooms that provided common supports. These common supports included the mathematics task, unpacking of the mathematics task, and an accompanying lesson plan with instructional goals. The assessment required teachers to lead a mathematics discussion in which students work on a mathematics task and participate in a discussion about their work. We turn next to describe each of these common supports.

**Fig. 2:**
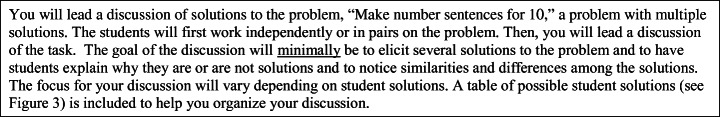
Mathematics task for the discussion

We selected a task that had been extensively piloted in elementary classrooms and can be found in existing materials (Ball & Shaughnessy, 2014). The task shown in Fig. 2 uses learner-generated examples (Watson & Mason, 2005; Watson & Shipman, 2008) to develop mathematics ideas, and it offers opportunities to compare and connect a range of solutions, strategies, or approaches, which represent one discussion structure (Kazemi & Hintz, 2014). The task was also selected because it focuses on mathematics content that is a key component of the elementary curriculum, can be used across grades, and because we conjectured that it could be implemented without necessarily being connected to the current instructional focus of a classroom. By specifying the mathematics task, we sought to control for the variation that would arise if teachers led discussions with tasks that are differentially suited for mathematics discussions. This was important for designing a tool that could be used to gather information about the skills of a group of PTs. Specifically, the task asked students to generate number sentences (equations) for 10 (e.g., 4 × 2 + 2 = 10).

**Fig. 3:**
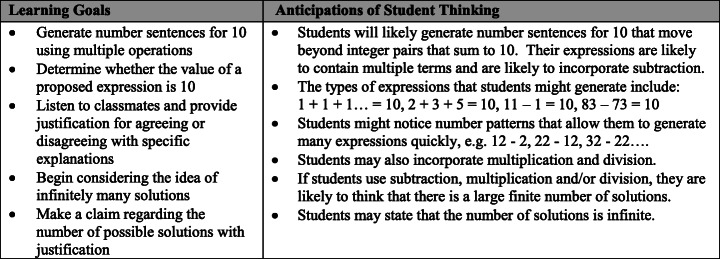
Learning goals and support for anticipating student thinking for grades 2 and 3

We also built in supports around other discussion-enabling practices. These included supports for understanding mathematics, anticipating student thinking (Smith & Stein, 2011), and adapting the task for different grades. Figure 3 shows an excerpt of the support materials for understanding the goals of the task and anticipating student thinking for middle elementary teachers. Supports also included a lesson plan with suggested timing, participation structures, and guidance for setting up the task. These supports were designed because discussion-enabling practices are critical to the work (e.g., Jackson et al., 2013). We wanted to ensure that the assessment would be useful across different points in a teacher preparation program in a way that focused on discussion-leading skills.

We used the decomposition of the teaching practice to develop a checklist that would support us in noticing the skills with leading a discussion that was being demonstrated by the PTs. There is an inherent tension between tracking teacher actions and student actions. Teacher actions provide insight into the moves that teachers can make; however, in the long run, these moves are ultimately only successful if students take up opportunities offered by those actions. To understand what teachers can do, we chose to focus our attention on the teacher’s moves even if students did not respond as intended. We focus on the learning opportunities offered by these moves to manage the tension between identifying teacher and student actions. This enables us to see what PTs can do to create such learning opportunities, whether or not they are taken up by students.

The goal of our checklist was to capture teachers’ skills with leading discussions. To capture their skills with carrying out a specific aspect of leading discussions, our checklist focuses on the teacher’s use of specific moves within the discussion-leading practices in our decomposition. In other words, moves are what we look for to see if the practice happens. For example, *launching* the discussion is one discussion-leading practice, and it has two associated moves in our decomposition. One move is to “direct attention toward the mathematics intended for the discussion.” We included this level of detail in the checklist to reflect our decomposition of practice and to meet the goal of developing an assessment that would allow us to capture detailed aspects of teachers’ discussion-leading skills. Importantly, we were assessing teacher skills, not student responses (e.g., students might not respond to a well-formulated question intended to probe their thinking). These teacher moves are identified in Table 1. Importantly, many of these teacher moves capture whether teachers are responsive to the ideas that children bring and how they participate in the discussion. For example, within *probing students’ thinking in relation to the mathematical goals*, we have included the move of “follows up on responses to make more student thinking about the mathematics available.” Within *orienting students to the contributions of their peers*, we have included “poses questions to students about others’ ideas and contributions.” The checklist was designed to be used in real time as teacher educators view a video of a discussion. We acknowledge that like the decomposition, the checklist for appraising performance is a living document, one that is intended to be expanded and refined by us and the larger mathematics education field over time, including revisions in response to the mathematical demands of a particular task. For example, the checklist could be used with a contextualized story problem and then teacher educators might want to see whether the PT supported the class in connecting solutions and representations to the problem context.

**Table 1:** Checklist for appraising performance

Move	Exemplar
Area of work: framing	
Launching	
Efficiently engages students in the mathematical work	*T:* “Can someone share one of their solutions”
Directs attention towards the mathematics intended for the discussion	*T:* “Can you share which operation(s) you used to make your number sentence for 10?”
Concluding	
Makes a closing statement that indicates the conclusion of the discussion.	*T:* “We are going to continue our discussion tomorrow, but we are going to stop for now”
Supports students in remembering or making sense of at least one key mathematical topic or practice	*T:* “Today in class we noticed similarities and differences in the number sentences for 10. We also concluded that there are infinitely many number sentences for 10”
Area of work: orchestrating	
Eliciting contributions	
Elicits multiple solutions, strategies, or ideas	Multiple solutions, strategies, or ideas are elicited from students during the course of the discussion*.
Elicits a range of student understanding or methods	Solutions, strategies, or ideas represent a range of potential understandings or methods*.
Engages several students in sharing their thinking	Several students share their thinking during the discussion*.
Probing students’ thinking in relation to the mathematical goals	
Poses questions to get students to explain their thinking about processes	*T:* “Can you walk us through what you did?”
Poses questions to get students to explain their understanding of key mathematical ideas	*T:* “Can you explain how you knew that process would make your equation equal 10?”
Follows up on responses to make more student thinking about the mathematics available	*T:* “Did everyone hear what was just said? That was a very important point to bring up, can someone restate what was just said?”
Provides support for students to complete their contribution or clarify their thinking	*T:* “I noticed you used parentheses, why do you think it was important to use parentheses?”
Orienting students to the contributions of peers	
Ensures that the class can hear others’ ideas	*T:* “Can you talk a little louder so everyone can hear you?”
Poses questions to students about others’ ideas and contributions, including asking students to comment on, add to, or restate another student’s idea	*T:* “Can someone state what was just said in another way?”
Supports the listening of the class through the use of moves that require all students to respond to others’ work	*T:* “Take a moment to write down whether you agree or disagree with what has been said and why?”
Encourages students to attend, listen, and respond to peers’ contributions in order to maintain productive and focused interaction	*T:* “Make sure you are paying attention to the person that is speaking”
Making contributions	
Uses moves such as redirecting, revoicing, and highlighting to keep the discussion on track	*T:* “That was a great point to mention and we will come back to it later in the discussion, but let’s focus on the different operations used to write number sentences.”
Ensures that substantive and relevant analysis is part of the discussion	*T:* “Did anyone notice any similarities or differences in the solutions that were shared?”
Makes mathematical contributions which enrich the core ideas of the discussion and keep the discussion focused on the learning targets	*T:* “Based on the solutions shared, how many number sentences for 10 can you make?”
Area of work: recording and representing content	
Records student ideas in ways that are true to the students’ contributions	Recordings are accurate representations of ideas shared by students*.
Attends to the accuracy of records and representations	*T:* “Do we need to add an equal sign to this expression to make it an equation?”
Records in ways that are clear, organized, and visible to the class	Records are clear and organized and appear to be visible to the class*.
Uses recordings which support student understanding and participation	Records have the potential to support student understanding and participation*.

*Due to the nature of these moves, we are unable to represent them succinctly using transcript lines.

In developing the checklist, we recognized that there are many ways to assess instruction, including rubrics that differentiate levels of performance in particular domains. We chose to use a checklist to record the presence or absence of particular moves with respect to a defined threshold. We were not tracking the prevalence of their use. A goal of using the tool to identify whether teachers were enacting moves in the different discussion-leading practices motivated this choice. Additionally, we built in a “not applicable (N/A)” choice because some specific moves may not be needed in particular cases. For example, students in a particular classroom might be used to talking to the class when sharing ideas and so the teacher might not need to prompt students to talk to the class as an orienting move.

### Present study

We made use of the tool we had developed to investigate whether an assessment could elicit and reveal details in teachers’ skills in leading discussions. The tool was intended to be used by a teacher educator as they watched a lesson live or via a video recording. The assessment was designed to be used with PTs enrolled in a teacher education program. However, to investigate these questions, we studied the discussion-leading practice of a group of first-year teachers (*n* = 9) from a range of teacher education programs because this enabled us to study the use of the assessment in different contexts.

## Methods

### Participants

The nine participants, all in their first year of teaching, spanned grades 1–5. All of the teachers were teaching in the USA, specifically in the state of Michigan. Contexts varied across urban, suburban, and rural schools. We recruited a diverse sample of teachers with respect to grade level, school district, and teacher preparation program. The purpose of this sampling was to gather data from a range of first-year teachers with different prior experiences who worked in different contexts; however, this sample was not intended to be representative of all first-year teachers. Although the teachers were graduates of a range of teacher education programs, all of the teachers reported that they had received instruction on leading mathematics discussions as part of their preparation program. All teacher names used in subsequent sections are pseudonyms.

### Data sources

Data sources for this study include a video record of one discussion for each participant. We provided the planning materials for the discussion (*Number Sentences for Ten)* a week before they were scheduled to teach the discussion and suggested that participants spend 45 min preparing. Teachers were aware that we were studying their discussion-leading skills and were provided with the areas of work being studied. Teachers were given suggested timing for the lesson––45 min, a typical amount of time for a mathematics lesson in elementary schools in the USA. Teachers determined for themselves how long the lesson should be in their own classrooms. This is analogous to how curriculum materials are used. A member of the research team video recorded the discussion, focusing on teacher and student interactions. The whole class discussions ranged from 15 to 45 min in length, which did not include independent work time on the problem before the launch of the discussion.

### Appraising performances

The analysis of the video data proceeded in two phases. In the first phase, the appraisal of the discussions was conducted by the research team by independently watching each video and using the checklist (see Table 1) to capture attributes of the performance. In the second phase, the team discussed cases where there were differences in observations on the checklist, referencing and refining a codebook as needed to reach consensus.

## What did the discussion assessment reveal about teachers’ discussion-leading skills?

We then examined the extent to which the assessment was able to reveal detailed aspects of teachers’ discussion-leading skills. The results are organized by the areas of work of leading a mathematics discussion. In presenting the results, we do not show an application of the tool to a transcript for two reasons. First, the tool was not intended to be applied to transcripts as the level of detail that is available in traditional transcripts does not fully support using the tool and because most teacher educators would be relying on watching a video or observing a lesson live rather than examining a transcript (for practical reasons). As a result, our coding work was done using the videos themselves (not transcripts). Second, because we were formatively assessing discussions, which were often 15–30 min long, it would be impractical to present the full transcript and a tool and to highlight the particular evidence that was used to classify a teacher as demonstrating (or not demonstrating) a particular skill. We do, however, include transcript lines as follows to illustrate particular points.

### Framing

Framing includes *launching* and *concluding* a discussion. We begin by noting the moves that teachers used to launch the discussion. Seven out of the nine teachers began the discussion with a prompt that “efficiently engaged the class in the mathematical work.” Eight out of nine teachers “directed the students’ attention towards the mathematics intended for the discussion in their launch.” We also looked at the moves that teachers used to *conclude* the discussion. Seven out of the nine teachers “made a closing statement.” In the cases where the teacher did not “make a closing statement,” the discussion often ended abruptly. Five out of the nine teachers “supported students in remembering or making sense of at least one key mathematical topic or practice from the discussion.” For instance, Mr. Houston ended the discussion by drawing on students’ ideas about whether they could ever finish finding number sentences and introducing the term infinity.

### Orchestrating

Orchestrating includes the discussion-leading practices of *eliciting contributions*, *probing students’ thinking in relation to the mathematical goals*, *orienting students to the contributions of peers*, and *making contributions*. Specific examples of what teachers did when they demonstrated the skill are indicated in Table 1. All of the teachers demonstrated skill with “eliciting multiple solutions, strategies, or ideas,” “eliciting a range of student understanding or methods,” and “engaging several students in sharing their thinking.”

We also looked at the extent to which teachers’ *probed students’ thinking in relation to mathematical goals*. Six out of the nine teachers “posed questions to get students to explain their thinking about processes.” However, not all teachers demonstrated such skill. For example, Ms. Valentino rarely asked students to explain their thinking, and students were not spontaneously justifying their thinking. Five out of the nine teachers “posed questions to get students to explain their understanding of key mathematical ideas.” Eight out of the nine teachers “followed up on student responses to make more student thinking about the mathematics available to the class.” Seven of the nine teachers “provided support for students to complete their contribution or clarify their thinking.”

Then we examined the moves that teachers made to *orient students to the contributions of peers*. One of the nine teachers explicitly made moves to “ensure that the class could hear others’ ideas” by asking students to speak loudly, and in another five cases, the students appeared to be consistently speaking loudly to the class without prompting. The remaining three teachers did not make such moves, and it appeared that students were unable to hear the contributions of peers at points. Six of the nine teachers “posed questions to students about others’ ideas and contributions, including asking students to comment on, add to, or restate another student’s idea.” Eight of the nine teachers “supported the listening of the class through the use of moves that required all students to respond to others’ work,” such as asking the class to show whether they agreed or disagreed with a number sentence using a “thumbs up” to agree or a “thumbs down” to disagree. Four of the nine teachers demonstrated the skill of “encouraging students to attend, listen and respond to peers’ contributions.” Such a move is important for maintaining productive and focused interactions. However, in two of the five cases where the teacher did not demonstrate the skill, the class was consistently engaged in productive and focused interactions and such a move did not seem necessary.

*Making contributions* is a strategic move that teachers can use in a discussion. All of the teachers demonstrated skills with “using moves such as redirecting, revoicing, and highlighting ideas in order to keep the discussion focused.” Seven of the nine teachers demonstrated the skill of “ensuring that substantive and relevant analysis is part of the discussion.” Four of the nine teachers “made a mathematical contribution that enriched the core ideas of the discussion and kept the discussion focused on the learning targets”––for example, naming the commutative property.

To illustrate some of the use of the moves within orchestration with particular attention to *orienting students to the contributions of their peers*, we turn to the case of Ms. Wheeler, a fourth-grade teacher, and we consider the middle of the discussion. At this point in the discussion, Ms. Wheeler had already “elicited multiple solutions,” “elicited a range of student methods,” and “engaged several students in sharing their thinking.” Ms. Wheeler named for the class that she saw that some of the students were using parentheses in their number sentences. Then, she invited Antonio to share a number sentence using parentheses.

Ms. Wheeler: I also saw some long numbers sentences in which people were using parentheses, like we learned last unit, in unit three. Antonio, you were the first one I noticed that had that. Would you like to show one of your sentences that had the parentheses, like that first one you had with the sevens, okay? Oh, please don’t call over friends. Antonio, you can write on the smart board on either side and let’s do the work along with him. I’m wondering how he’s going to get his answer using a parentheses.

Antonio: *Writes*:

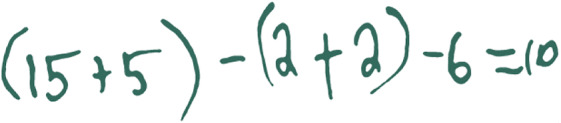


After Antonio wrote his number sentence on the board, Ms. Wheeler asked him to read the number sentence aloud to the class.

Ms. Wheeler: Okay. Will you read in that nice strong voice for us?
Antonio: Fifteen plus 5 minus 2 plus 2 minus 6 equals 10.

Next, Ms. Wheeler acknowledged that Antonio had already justified his reasoning to her and invited another student to try to explain Antonio’s thinking (“poses questions to students about others’ ideas and contributions, including asking students to comment on, add to, or restate another student’s idea”).

Ms. Wheeler: Okay. Now, Antonio, when I came over to you, you explained to me how you got your answer. I’m wondering if now a different friend can explain why Antonio’s answer, if they believe that yes it’s an answer? So I see some friends with thumbs up. Go ahead and try and jump up and explain why 15 plus 5 in parentheses, minus 2 plus 2 in parentheses, minus 6 would be equal to 10. You want to try and explain, Sophia?

Sophia: Yeah, because so 15 plus 5 is 20 and 2 plus 2 is 4. And so 20 minus 4 is 16, and 16 minus 6 is 10.

Ms. Wheeler went on to ask whether the class and agreed with Sophia’s proof of Antonio’s answer (“supports the listening of the class through the use of moves that require all students to respond to others’ work”).

Ms. Wheeler: Ah. Do we agree with Sophia’s proving there? Proving Antonio’s answer? Antonio, is that what you were thinking when you did it?

Antonio: Yeah.

As Ms. Wheeler asked the question, students used a hand signal in the class that indicated agreement. While she did not explicitly ask students to show their agreement/disagreement through a hand signal, such a norm appeared to be in place as students used a hand signal to indicate agreement. Ms. Wheeler then went on to invite Alex to share. In her request she is attentive to “ensuring that the class can hear others’ ideas” and “encourages students to attend, listen and respond to peers’ contributions in order to maintain productive and focused interactions*.*”

Ms. Wheeler: Wonderful, wonderful. Thank you, thank you. And last but not least I saw Alex, you had some written with parentheses as well, didn’t you? Would you be willing to share one of your ones with parentheses? Okay, good for you. Let’s watch Alex and see if we can figure out how he’s thinking about this. Let’s watch. All right, can you read your sentence in a nice, loud voice, Alex?

Alex: Well, I got that, 7 plus 7 equals 14 minus 4 equals 10. I know it’s because when you-- if you add 7 with 7, equals 14.

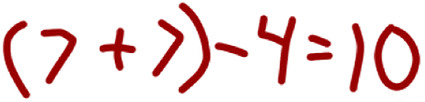

Ms. Wheeler: Right.

Alex: And, well, I thought that maybe if 7 plus 7 equals 14, what number will you subtract that would give you 10. So I thought that was 4. And then it gave me the answer.

Ms. Wheeler: Interesting, interesting. Do we agree with Alex? Show me your discussion signs, thumbs in the air, what do you think? Okay, oh I see some agreeing. Does anyone want to try to rephrase what Alex said and put it in their own words, why his would be equal to 10? Why do you think that that one would be equal to 10? You could share similar strategies, add something you thought about differently.

Alex shared his solution and reasoning with the class. Ms. Wheeler then went on to invite the class to indicate whether they agreed or disagreed with Alex (his solution) using discussion hand signals (“supports the listening of the class through the use of moves that require all students to respond to others’ work”). The class expressed agreement. Then Ms. Wheeler invited students to restate Alex’s solution, to add on, or to share how they thought about something similarly or differently (“poses questions to students about others’ ideas and contributions, including asking students to comment on, add to, or restate another student’s ideas”). In this short segment of the discussion in Ms. Wheeler’s classroom, we see evidence of her skills with all four of the moves related to *orienting students to the contributions of peers*.

Ms. Barber’s fourth-grade classroom offers a contrasting case in terms of demonstrated skill with moves related to *orienting students to the contributions of peers*. Ms. Barber started the discussion by contributing one solution (2 + 8 = 10). Then Ms. Barber invited a student (Mackenzie) to share a solution. Ms. Barber recorded the solution on the board.

Mackenzie: I did 6 times 2 minus 2 equals 10.

Ms. Barber: Oooh! 6 times 2 – did you put parentheses?

Mackenzie: Yeah.

Ms. Barber: Okay. Minus what?

Mackenzie: Minus 2.

Ms. Barber: Minus 2 equals 10.

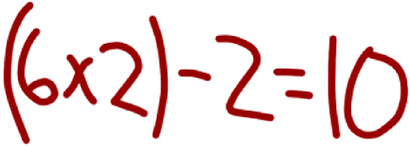

Ms. Barber asked questions to clarify whether Mackenzie had used parentheses. Importantly, these questions seemed tied to represent Mackenzie’s solution on the board rather than *probing student thinking in relation to the mathematical goals*. After representing Mackenzie’s strategy, Ms. Barber did not press Mackenzie about her reasoning nor did she engage in moves to *orient students to the contributions of their peers*. Instead, Ms. Barber commented that she is seeing a pattern in the solutions shared thus far and invited another solution.

Ms. Barber: So we’re noticing a little bit of a pattern. Who else can give me another equation? David? You can give me one.

David: 9 plus 1.

Ms. Barber: 9 plus 1. What else do we have with it?

David: Equals 10.

After David shared, Ms. Barber does not follow up on his solution to probe his thinking or to orient students to the contributions of their peers. Instead, she emphasized the meaning of the equals sign in an equation, invited students to write additional solutions on the board (without talking about the solutions), and then sent students to work with a partner to generate additional solutions. Across the discussion, Ms. Barber did demonstrate skill with “eliciting multiple solutions,” “eliciting a range of student methods,” and “engaging several students in sharing their thinking”; however, she did not demonstrate skill with any of the moves related to *orienting students to the contributions of their peers*.

### Recording and representing content

We examined the use of four specific moves for *recording and representing content*. First, we found six of the nine teachers “recorded student ideas in the public space in ways that were true to the students’ contributions.” Two teachers had students do all of the recordings independentl7y; thus, we were not able to see these teachers’ skills with this move. One teacher repeatedly recorded student ideas in ways that did not match student contributions. Second, four of the nine teachers “attended to the accuracy of the records and representations” on the board. In other cases, the teachers allowed inaccurate number sentences to be listed on the board (e.g., 7 + 4 = 10) at the end of the discussion with no annotation. In other words, the class had not assessed the accuracy of the solutions recorded, and the teacher did not bring attention to the inaccuracies. Third, five of the nine teachers “recorded on the board in ways that were clear, organized, and visible to the class.” For two teachers, the recordings made by the teacher did not meet these criteria. In the two cases where students recorded, the recordings had different qualities. In one case, the recordings made by students were clear, organized, and visible, and so we coded it as “not applicable,” but this was not the case for the other teacher. Finally, we found that only five of the nine teachers “used the recordings to support student understanding and participation in the discussion.”

### Detailed aspects of teachers’ skills

We found that the assessment and accompanying checklist revealed detailed aspects of teachers’ skills. To illustrate how they provided such information, we describe what the checklist revealed about the performance of one fifth-grade teacher. Mr. Weber launched his discussion efficiently and framed the mathematics to be discussed, although he did not have all the students’ attention before beginning. Within the practice of eliciting, Mr. Weber enacted all of the moves we sought to capture, including eliciting multiple solutions/strategies. He demonstrated less skill in probing and orienting, posing no questions that probed students’ processes, or understanding and doing little to orient students to each other’s contributions. He reminded students to listen to each other and asked for signs of agreement/disagreement with a contribution, but he did not ask students to engage in other ways with others’ ideas. Mr. Weber did enact moves within the practice of making contributions. He revoiced and asked questions focused on the key ideas; however, his mathematical contributions did not enrich the core ideas of the discussion nor did they keep the focus on the learning goals. Throughout, he demonstrated skills with recording and representing content as he recorded students’ number sentences on the board, although he did not always attend to the mathematical accuracy of his records. Mr. Weber concluded by taking stock of the discussion and worked to support students in remembering that they had determined that there could be infinitely many solutions to the task.

## Discussion

Having access to detailed information about PTs’ developing proficiencies with teaching practices is crucial for quality teacher education. Assessments must make it possible for teachers to demonstrate their skills in authentic ways. Moreover, teacher educators must be able to use the data gathered from such assessments to focus efforts to support PTs’ development. This study sought to examine the utility of using an assessment to assess teachers’ discussion-leading practice for formative purposes. The assessment was accompanied by a set of supports for teachers, which included the mathematics task and supports for understanding the mathematics of that task, anticipating student thinking, and adapting the task for different grades. A lesson plan with suggested timing, participation structures, and guidance for setting up the task was included. This enabled the assessment to focus squarely on teachers’ enactments. The study findings reveal that the assessment prompted, and the checklist captured, useful details about teachers’ skills. For example, the data captured useful information about the group of teachers that could be used to design subsequent learning experiences. In particular, this group of teachers would benefit from support focused on attending to the accuracy of the records and representations on the board as only four of the nine teachers did so, which meant that five of the nine teachers allowed inaccurate records to remain on the board without correction. It also provides useful information for supporting an individual teacher. In the case of Mr. Weber, he asked students whether they agreed/disagreed with others’ ideas but could benefit from opportunities to build skills by encouraging students to engage with others’ ideas. In this way, the assessment provided information that could be used to support future work with teachers.

We identify four key limitations of the design and use of the assessment and checklist. First, we recognize that the assessment and tool are grounded in a particular decomposition of the practice of leading a discussion that aligns with particular discussion structures and impacts what can be seen. Our assessment is designed to gather information about teachers’ discussion-leading skills in a problem-based classroom scenario with a focus on teacher moves. A second limitation concerns the use of threshold statements for our checklist, yielding information primarily about the presence or absence of each move within a practice rather than the use of a rubric, distinguishing the quality or quantity of teachers’ enactments above the threshold. However, teacher educators need a tool that yields useful information for PTs and teacher educators that they can use as they watch videos of PTs’ teaching. Checklists allow for in-the-moment scoring, whereas a rubric requires that a teacher educator watch the entire performance and often rewatch it before making a judgment. Further, the checklist addressed our goal of capturing fine-grained details about the performance of a PT (e.g., whether they could carry out a specific move). Thus, despite the limitations of the checklist, we believe that it is supportive of the work of teacher education.

A third challenge is the potential interaction of content knowledge for teaching and discussion-leading practice. Even with standardization of the mathematical task, the focus on content that matters for teaching mathematics, and supports built into the materials, there were instances in which content knowledge appeared to be a factor in the enactment. This is unsurprising as content knowledge for teaching is clearly intertwined throughout the work, particularly in probing, orienting, contributing, and recording. In these cases, it was unclear whether we were accurately capturing a teacher’s skill with particular moves of discussion leading or whether their use of content knowledge in teaching prevented them from demonstrating their discussion-leading skill. This is an inherent problem in examining teaching practice.

A fourth limitation is that we do not know whether the teachers in this study would perform differently if they enacted the discussion in a different context. The context could be interpreted along multiple dimensions, including, but not limited to, the mathematics task used, the grade level of the students, the student’s familiarity with participating in discussions, and the point in the school year. For example, if we had chosen a different sort of task, a contextualized task, we could have seen whether teachers could connect to student’s multiple mathematics knowledge bases (Turner et al., 2012). This is an inherent problem; however, because we are not making claims about teachers’ skills more broadly, we argue the assessment reveals important information about teachers’ skills with a discussion that can be used for formative purposes.

This study provides an important contribution to thinking about assessing PTs’ skills with practices of teaching. We found that it was possible to design an assessment for leading a discussion. This assessment accomplished many of our design goals. Its ability to capture a range of skills could make the assessment and checklist useful in teacher education. We believe that teacher educators could use such assessments to track PTs’ growth over time and to identify areas of strength and weakness concerning the practice. This would allow for targeted feedback and support of individuals and program-level curriculum design. The mathematics task would shift, but the checklist could be used across the assessments over time. The use of scaffolds for particular parts of a lesson also proved promising, narrowing the focus of the assessment on a single instructional practice.

Additionally, providing the mathematical task allowed for comparison across performances, as well as focusing the assessment squarely on discussion-leading practices through providing common supports. Further, common mathematics content has the potential to support the efficient and manageable use of the assessment and checklist. A common challenge in the USA is that when PTs select their own task, they all select different mathematics tasks. Elementary teacher educators are often working with more than twenty preservice teachers and if teacher educators are watching discussions of twenty discussions with different tasks, there is a significant time investment in unpacking each task before watching each discussion. Providing the mathematical task streamlines the work to assess performances and enables teacher educators to notice something about a group of PTs with respect to leading a discussion around a particular type of task.

Future work could systematically explore PTs’ use of the checklist to examine their own teaching practice and/or to provide feedback to a peer. The tool has the potential to support PTs in noticing the types of moves made in a discussion and the impact of those moves on students’ participation and learning in a discussion. Further, PTs could use the observation of the absence of a particular move as an opportunity to reflect whether there were missed opportunities in a discussion or whether the discussion, as it unfolded, did not require the move and why. In this way, it might be possible to encourage PTs to deepen their intentionality with using particular moves in teaching, understanding the impacts of the moves on students’ opportunities to learn, including the development of students’ mathematical identities. Further, the checklist could help mitigate the challenges associated with novices giving each other feedback, including potentially reinforcing teaching practice that does not serve students well, if accompanied by a set of guiding questions that press PTs to consider the impact of moves. While we have done such work in our own practices as teacher educators, we have not systematically explored the impact of using the tool in this way on PTs’ teaching practice over time.

We close by acknowledging that we view a decomposition of teaching practice as a “living” document. It is a framework for guiding work with teachers that will need refinement over time. We concur with Jacobs & Spangler, 2017) argument that it is not about having the “right” decomposition but rather that decomposition can support the development of common language, the teaching of teaching practice, and the formative assessment of teaching practice. As we use this decomposition, we continue to refine it with particular attention to infusing attention to teachers’ moves to disrupt patterns of inequity (i.e., attending to who is participating and intentionally inviting often marginalized voices into the conversation). Within each of the areas of work, we are also expanding consideration of supports that are needed for language learners in a discussion. As such, when others use the decomposition (and corresponding checklist), we intend for them to refine the decomposition (and corresponding checklist) based on their own contexts and growing research in the field.
